# An Assessment of the Impact of Urbanization on Soil Erosion in Inner Mongolia

**DOI:** 10.3390/ijerph15030550

**Published:** 2018-03-19

**Authors:** Li-Yan Wang, Yi Xiao, En-Ming Rao, Ling Jiang, Yang Xiao, Zhi-Yun Ouyang

**Affiliations:** 1 Research Center for Eco-Environmental Sciences, Chinese Academy of Sciences, Beijing 100085, China; wangliyan15010@gmail.com (L.-Y.W.); xiaoyang180317@sina.com (Y.X.); zyouyang@rcees.ac.cn (Z.O.); 2 College of Resource and Environment, University of Chinese Academy of Sciences, Beijing 100049, China; 3 College of Geography Resource Sciences, Sichuan Normal University, Sichuan 610101, China; enmingrao@sohu.com; 4 Zhongshan Research Institute of Environmental Protection Science Co., Ltd, Guangdong 528403, China; lingiang@aliyun.com

**Keywords:** assessment, Inner Mongolia, soil erosion, urbanization

## Abstract

Inner Mongolia, an autonomous region of the People’s Republic of China, has experienced severe soil erosion following a period of rapid economic development and urbanization. To investigate how urbanization has influenced the extent of soil erosion in Inner Mongolia, we used urbanization and soil erosion data from 2000 through 2010 to determine the relationship between urbanization and soil erosion patterns. Two empirical equations—the Revised Universal Soil Loss Equation (RUSLE) and the Revised Wind Erosion Equation (RWEQ)—were used to estimate the intensity of soil erosion, and we performed backward linear regression to model how it changed with greater urbanization. There was an apparent increase in the rate of urbanization and a decrease in the area affected by soil erosion in 2010 compared to the corresponding values for 2000. The urban population stood at 11.32 million in 2010, which represented a 16.47% increase over that in 2000. The area affected by soil erosion in 2000 totaled 704,817 km^2^, yet it had decreased to 674,135 km^2^ by 2010. However, a path of modest urban development (rural–urban mitigation) and reasonable industrial structuring (the development of GDP-2) may partially reduce urbanization’s ecological pressure and thus indirectly reduce the threat of soil erosion to human security. Therefore, to better control soil erosion in Inner Mongolia during the process of urbanization, the current model of economic development should be modified to improve the eco-efficiency of urbanization, while also promoting new modes of urbanization that are environmentally sustainable, cost-effective, and conserve limited resources.

## 1. Introduction

Urbanization is an inevitable trend in humanity’s development, and is an important symbol of the progress made in science and technology. More than half of the world’s human population now lives in urban areas, confirming that the world has now entered the urban society age [1]. This shift has also occurred in China [2,3,4]. A key aim of the Communist Party of China is to build sustainable cities, those characterized by intensive, intelligent, and green design. In general, a sustainable city sustains the welfare of its people without jeopardizing its capacity to maintain and improve its ecosystem services [5]. Urbanization can be defined as a concentrated human presence in a residential and industrial setting and its associated affects [6,7]. Importantly, the urban extent of most metropolitan areas is expanding into adjacent rural landscapes [8,9,10]. It is well known that urbanization leads to substantial changes in human society, such as the promotion of economic development, the expansion of an urban area, and an improvement in life’s material conditions [11]. However, urbanization can also drive many environmental issues and problems, such as climate change [12,13], environmental pollution [14] and loss of agricultural productivity [15,16,17], while also increasing the population’s exposure to major risk factors for disease, especially those linked to the deteriorating environmental conditions [18]. Soil erosion is a major environmental problem throughout the world, including China [19]. Nearly one-third of China’s land suffers from soil erosion [20]. Direct consequences of soil erosion can severely impact the economy, environment and human health, not only in the local area but also in downwind areas. From an economic perspective, it can cause traffic accidents and airport closures, harm food crops [15,16,17], and lead to great financial loss [21]. From the environmental perspective, dust particles carrying many pollutants (i.e., ammonium ions, nitrate ions, and heavy metal compounds and so on) are blown into the atmosphere, resulting in poor air quality [22,23]. From the human heath perspective, dust storms make many people feel uncomfortable, which prompts them to go to hospitals to treat associated eye and respiratory system ailments [24].

Inner Mongolia is located in the north of China, where industrialization is highly concentrated and the pace of development is fast [25,26]. Urbanization can significantly change land use types and their associated ecosystem services. Inner Mongolia suffers from both water erosion and wind erosion [18,19,20] because of its arid and semi-arid territorial climate, uneven evaporation and rainfall regimes, strong winds and large area covered in sand. The central-western region of Inner Mongolia was a major dust source area fueling the strong sandstorm that affected China, Korea, Japan, and the United States [27,28,29]. Due to its ecological vulnerability, the continued ecological functioning of Inner Mongolia is important for China’s future. Therefore, further urbanization in this region ought to unfold in a sustainable way to reduce the threat of soil erosion and thereby guarantee the key ecological functions. To this end, our study’s results may provide useful scientific advice to inform the sustainability of urban development and the restoration of its ecological environment; they should help to establish a scientific pathway toward maintaining harmony between humans and the environment and safeguard public health during the rapid urbanization process.

## 2. Study Area

**Figure 1:**
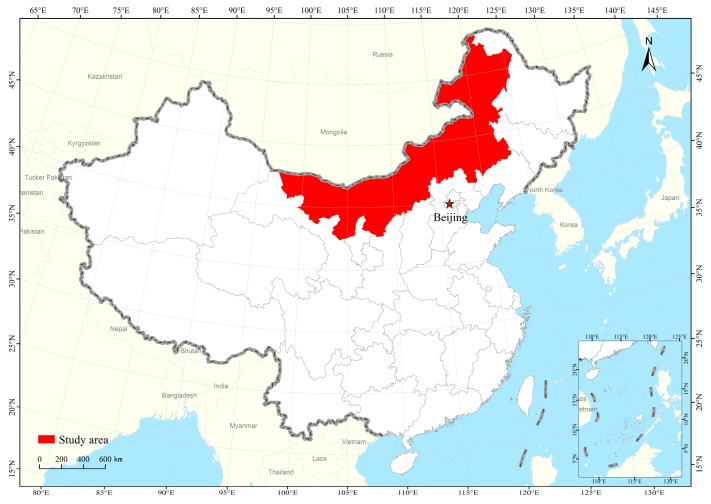
Location of Inner Mongolia.

Inner Mongolia lies in northern China and covers an approximate area of 1,144,900 km^2^, spanning 1700 km from south to north and 2400 km from east to west, and has 12 prefecture-level cities (Figure 1). The area adjoining Mongolia and Russia is also the energy production and animal husbandry production base of northern China. In terms of its geographic coordinates, Inner Mongolia’s longitude is 97°–126° east, and its latitude is 37°−53° north. The climate here is arid and semi-arid, with annual average temperature of −1 °C to 8 °C. The winter in Inner Mongolia is long, cold and dry, with an average temperature of −3.5 °C to 15 °C, and the summer is short, with an average temperature of 20.1 °C to 25.3 °C. The precipitation varies greatly from 50 mm in the west to more than 450 mm in the east. The average number of strong wind days ranges from 10 to 40 days per year, with 70% of them occurring in the spring. The main soil types are black, dark brown, brown, sierozem soil, and grey-brown desert soil [30]. Land use varies across the region geographically. Inner Mongolia can be divided into three aspects: east Inner Mongolia is characterized by large and extensive forest, while grassland dominates in the central parts and desert in the west. Crucially, Inner Mongolia is recognized as one of China’s most important ecological barriers against sandstorms and soil loss to erosion. The region is known to play a vital role as a barrier in northern China for providing soil retention and sandstorm prevention to the local and downstream people who suffer from water erosion and dust storms [30].

In 2010, the population of Inner Mongolia reached 25 million people, which includes both the its urban and rural populations. From 2000 to 2010, Inner Mongolia underwent rapid economic development and urbanization with the establishment of many factories, mining area, railways, and other heavy construction projects.

## 3. Materials and Methods 

There are two classical empirical methods by which to estimate soil erosion, which is defined as the mass of soil lost per unit area and time: the Revised Universal Soil Loss Equation (RUSLE) [20,31,32,33,34,35] and the Revised Wind Erosion Equation (RWEQ) [30,31,36,37,38]. Using the RUSLE model and the RWEQ model in conjunction with a geographical information system (GIS) approach has proven powerful for estimating soil erosion. The RUSLE model is described as follows:

*A* = *R* ⋅ *K* ⋅ *LS* ⋅ *C* ⋅ *P*, (1)

where *A* is the mean annual soil loss rate (t ha^−1^ year^−1^), *R* is the rainfall erosivity factor (MJ ha^−1^ mm^−1^ year^−1^), *K* is the soil erodibility factor (t MJ^−1^ mm^−1^), *LS* is the topographic factor comprising the slope length factor (*L*) and slope steepness factor (*S*), *C* is the cover management factor, *P* is erosion control practice factor. *C* and *P* are dimensionless.

Rainfall erosivity factor (*R*) represents the potential ability of rainstorms to induce soil erosion [31,39]. In this paper, we adopted the daily rainfall erosivity model [31,40], by using rainfall data collected from 603 weather stations from 1980 to 2010. Interpolation using the Kriging method was then relied upon to obtain a raster layer of the R factor (at a spatial resolution of 90 m).

Soil erodibility factor (*K*) reflects the sensitivity of soils to erosion, which is closely related to the attributes of soils [39].The erosion/productivity impact calculator [31,41] was used in calculations of the soil map and soil attribute data.

Topographic factor (*LS*) refers to the influences of terrain features (i.e., *L*-slope length, *S*-slope steepness) on soil erosion [42]. We integrated the relevant research to date on gentle slopes and steep slopes, and performed our calculations using different slope segments [20,31,32]. A digital elevation model (DEM) was used in this model.

Vegetation cover factor (*C*) presents the effects of different land types on soil erosion. The Vegetation cover factor (*C*) were assigned referred to relevant studies [20,32].

Erosion control practice factor (*P*) is the ratio of soil loss with a specific support practice. In this study, *P* is assigned to one because of the absence of conservation support practices data.

The RWEQ model can be expressed as follows:

*S_L_* = (2*Z*/*S*^2^) ⋅ *Q_max_*^(*z/s*)2^, (2)

*Q_max_* = 109.8(*W_F_* ⋅ *E_F_* ⋅ *S_CF_* ⋅ *K*′ ⋅ *C*), (3)

*S* = 150.71 ⋅ (*W_F_* ⋅ *E_F_* ⋅ *S_CF_* ⋅ *K*′ ⋅ *C*)^−0.3711^. (4)

Here, *S_L_* is the rate of soil loss caused by wind erosion (kg m^−2^), *Q_max_* is the maximum transport capacity (kg m^−1^), *S* is the critical field length (m), *W_F_* is the weather factor (kg m^−1^), *S_CF_* is crusting factor, *E_F_* is the erodible fraction, *C* is the vegetation factor, and *K*′ is the surface roughness factor.

Weather factor (*W_F_*) represents the influence of climate conditions(i.e., wind, snow cover, soil moisture and so on) on wind erosion [30]; *W_F_* is determined by dividing the total wind value for each period by 500, then multiplying that by the number of days in the period [31]. 

Soil erodible factor (*E_F_*) corresponds to that fraction of the surface 25 mm of sand or soil that is smaller than 0.84 mm in diameter, as determined by a standard compact rotary sieve [31,43] .The developed formula is cited by the relevant researches [30,31].

Soil crusting factor (*S_CF_*). When raindrops land on the surface of soil, there is a redistribution of soil particles and the formation of surface crust [30,31]. The resulting soil surface can be extremely hard or very fragile, and it may decrease or increase the wind erosion potential [30,31,44]. The formula is referenced to the relevant researches [30,31].

Vegetation factor (*C*). In the RWEQ, three parts of land surface are considered: non-erodible plant material (flat residues), the plant silhouette from standing plant residues (standing residues), and growing crop canopies (crop canopy) [45]. The detail formula is referenced to relevant studies [30,31].

Surface roughness factor (*K*′). This *K*′ value was calculated using the Smith–Carson equation [30,31].

Results were divided into five categories: slight (200–2500), moderate (2500–5000), intense (5000–8000), strong (8000–15,000) and severe erosion (>15,000), according to the national industrial standard of Classification Standard of Soil Erosion [46]. The unit of expression of soil erosion is t km^−2^ year^−1^.

This investigation relied on existing data from the Chinese Academy of Sciences (CAS), the Inner Mongolian government, and other scientific research institutions. Details concerning these data sources are summarized in Table 1.

## 4. Results

### 4.1. Urbanization

Urbanization is arguably the most dramatic form of human social progress. Although urbanization occurs throughout China, it is especially prevalent in Inner Mongolia. Urbanization is generally measured by the percentage of population living in urban areas [47], a massive expansion in urban infrastructure and economy of a city [48]. This view also applies to Inner Mongolia, for which we analyzed the spatiotemporal processes leading to urbanization and its change over time. The results revealed that in 2010, the total population of Inner Mongolia was 23.06 million, 45.29% of which was urban. This indicated that Inner Mongolia was in the rapid development stage of urbanization according to the theory of international development of urbanization [49]. Compared with 2000, the level of urbanization in 2010 had increased, with the urban population experiencing 16.47% growth over the ten-year period. This was much higher than the rate of growth in either the total or rural populations (Table 2).

The development of the economy was another important factor in the process of urbanization. Gross domestic product (GDP) in 2010 was 11,438.67 billion yuan, which represented an increase of 809.89% over the ten-year period studied. The GDP-1 (primary industry) decreased by 64.87% over this time whereas the GDP-2 (secondary industry) increased. The most obvious change over the study period was the inevitable expansion of the urban area which grew from 9800 km^2^ to 12,900 km^2^ (Table 2). In the process, much land that was previously forest, grassland, and arable land was lost due to urban expansion.

### 4.2. Land Use Matrix

The land use change matrix may be described as the changes in the characteristics of regional land use structure over a certain period of time. It can be used to explain the conversion of different land types during a given study period. Here, we assessed the dynamics in land use between 2000 and 2010 and found a decrease in grasslands (3128.68 km^2^) and wetlands (934.21 km^2^) and an increase in urban lands (2444.61 km^2^) and forests (930.15 km^2^). The total loss in grasslands were mainly replaced by urban land (1744.03 km^2^), forest (775.63 km^2^) and farmland (567.50 km^2^). With regard to farmland, it underwent a net increase of 281.26 km^2^: 1100.16 km^2^ of farmland was converted to urban land (432.60 km^2^) and natural vegetation (i.e., forests, wetlands: 385.26 km^2^), but there was 1381.41 km^2^ of newly reclaimed farmland at the expense of grasslands (679.65 km^2^) and wetlands (568.77 km^2^) (Table 3).

### 4.3. Soil Erosion

**Figure 2:**
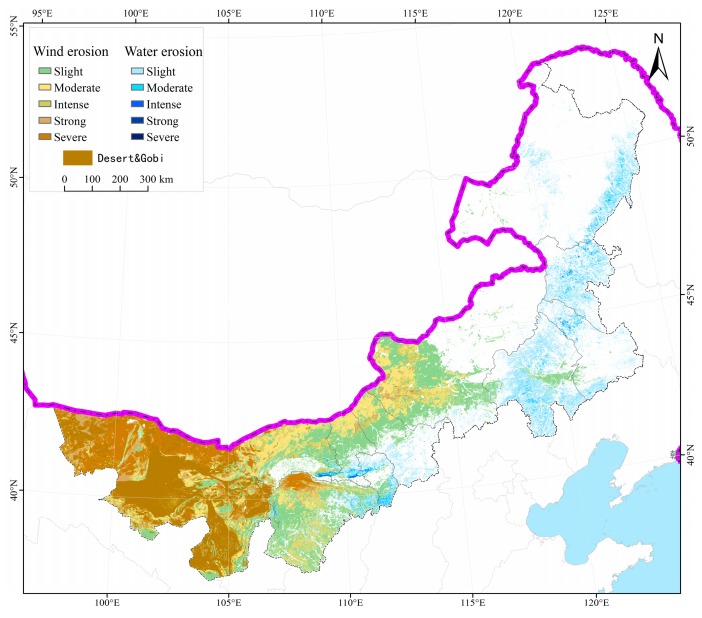
The distribution of soil erosion in 2010 in Inner Mongolia.

Soil erosion in Inner Mongolia had been reduced in recent years, according to the results of this study. Figure 2 shows the extent and severity of soil erosion in 2010. The total area of land with soil erosion was 674,135 km^2^, with water erosion accounting for 124,824 km^2^ and wind erosion accounting for 599,546 km^2^. The areas affected by water erosion were mainly in the wetter eastern parts of the region, while those areas impacted by wind erosion were in central-western regions.

**Figure 3:**
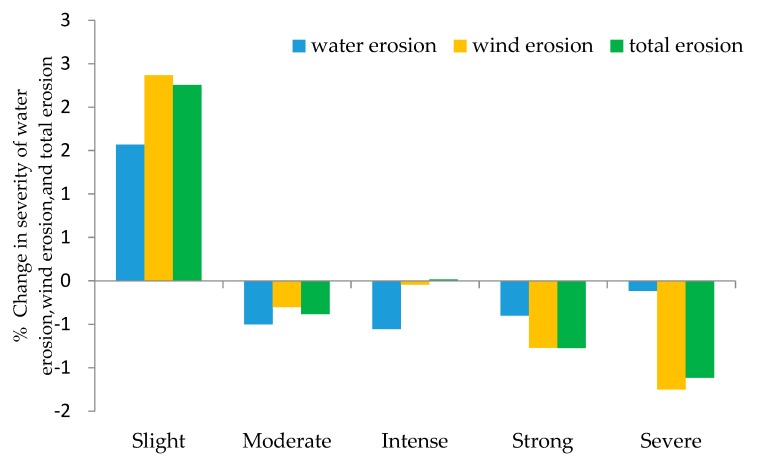
Changes in areas of different categorical intensities of soil erosion, in proportion to all erosion areas for the period of 2000–2010.

Through a comparative assessment of soil erosion extent in the years 2000 and 2010, it was clear that soil erosion by water and wind had decreased in Inner Mongolia (Table 4). The overall area affected by soil erosion was reduced from 704,817 km^2^ in 2000 to 674,135 km^2^ in 2010, which represented a decrease of 4.35%. The total area affected by water erosion decreased from 130,641 km^2^ in 2000 to 124,824 km^2^ in 2010 (a 4.45% reduction) while that by wind erosion decreased from 626,844 km^2^ in 2000 to 599,546 km^2^ in 2010 (a 4.35% reduction). In terms of five categorical intensities of erosion, only areas of slight erosion had increased in extent between 2000 and 2010. By contrast, the areas of severe and strong erosion were reduced in extent by 13.88% and 9.05%, respectively (Table 4). Between 2000 and 2010, the severity of soil erosion that did occur reduced, with areas that experienced high intensity erosion in 2000 only experiencing low intensity erosion in 2010 (Figure 3). Evidently, low intensity erosion, in the form of slight erosion, still plays an important role in the soil erosion dynamics of Inner Mongolia because of its extensive distribution across the region. The proportion of slight erosion in total area affected by soil erosion increased from 46.64% in 2000 to 48.89% in 2010, representing a growth of 2.26%. This included increases in slight erosion in the area affected by water erosion (from 81.83% in 2000 to 83.40% in 2010), and an increase in the area affected by wind erosion from 37.33% in 2000 to 39.70% in 2010 (Figure 3).

### 4.4. The Relationship between Urbanization and Soil Erosion

**Figure 4:**
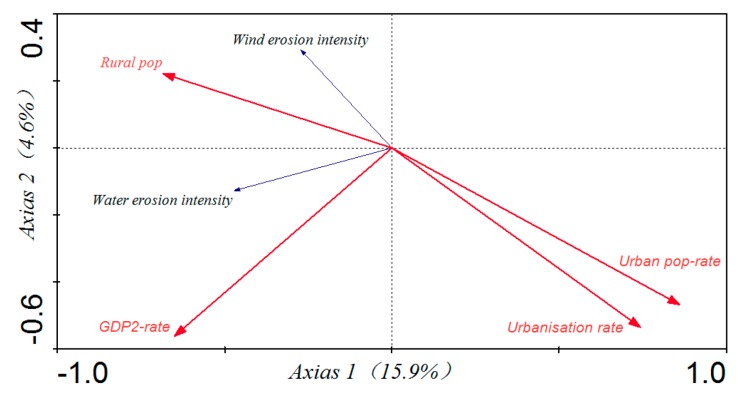
The integrated interpretation of urbanization on soil erosion in Inner Mongolia.

We used bivariate correlation analysis to test the relationship between urbanization and soil erosion. All these results were summarized in Table 5, in which soil erosion is separated into the contributions from wind and water erosion. The change in water erosion intensity over the ten-year period was positively correlated (*p* < 0.05, *p* < 0.01) with change in GDP-2 rate and the rural population, and negatively correlated (*p* < 0.05) with the change in urban population and urbanization rate. The change in area affected by water erosion was strongly positively correlated with change in rural population yet significantly negatively correlated with the change in urban population rate that had become urbanized. Similarly, the change in the wind erosion intensity was significantly negatively correlated (*p* < 0.01) with the change of urban population and the rate of urbanization, and significantly positively correlated (*p* < 0.05) with the change in the rural population. The relationships between the change in GDP-2 rate and changes in both the water and wind erosion intensities were negatively correlated (*p* < 0.05, *p* < 0.01). The total variance in soil erosion explained by urbanization was 20.50% in Inner Mongolia and the percent variance explained by each single factor was ranked in this order: urban population (12.7%) > urbanization rate (10.10%) > GDP-2 rate (8.1%) > rural population (7.7%) (Figure 4).

## 5. Discussion

Soil erosion is a surface process that is often accelerated by human activity. Between 2000 and 2010, the level of urbanization in Inner Mongolia had increased significantly, along with an increase in urban population, a decrease in rural population, an increase in GDP-2, and an expansion of the overall urban area. Soil erosion problems clearly declined in the ten-year period examined. A similar trend—of rapid urbanization and reduced soil erosion—obtained here was reported in Inner Mongolia [30]. Indeed, urbanization is known to have a positive influence on sensitive arid and sub-arid regions [51,52]. The growth of the total human population, especially of its rural population, put more pressure on agricultural land. About 679.65 km^2^ of grassland and 568.77 km^2^ of wetland were converted to farmland in Inner Mongolia from 2000 to 2010. The phenomenon has led to the acceleration of land desertification resulting in the increased risk of soil erosion [30,53,54,55]. It has been reported that with the development of croplands, the potential soil erosion increases by an estimated 17% [56]. Similarly, a potential overall increase in global soil erosion is driven by farmland expansion [57]. Without the targeted management of farming activities and well-planned conservation measures, an increase in the rural population could indirectly accelerate soil erosion [55,57]. By contrast, with an increase in the urbanization rate, more and more people leave rural life for the cities, where they become concentrated and engaged in non-agricultural work instead of prior agricultural activities (e.g., planting and grazing). This process could be called “eco-migration”, carried out from 1998, to reduce disturbance to soil and thus provide a favorable environment for natural ecosystem restoration to proceed [58,59]. Approximately 1427.93 km^2^, 659.94 km^2^ area of grassland and farmland, respectively, were restored to forest and wetland (Table 1).

From the perspective of demand, the economy’s development has placed enormous pressures on the scarce natural resources of Inner Mongolia, and this extensive economic development has led to greater demands for energy and resources, which is characterized by high energy consumption, large amounts of waste, and serious levels of pollution. From 2000 to 2010, about 2469.73 km^2^ area of other land was converted to urban land, the GDP-2 rate had improved by 29.66% and the GDP-1 rate had fallen by 64.87%; together, this indicates that the economic structure has been transformed, shifting in emphasis from agriculture to industry. The production of energy and the mining of mineral resources have been accompanied by the destruction of vegetation and the accumulation of waste materials [60]. Large areas of grassland, approximately 1754.03 km^2^ had been converted to urban land via transportation and excavation that have markedly disturbed local natural ecosystems. The soil erosion generated in just one year due to this activity would be the equivalent of the amount of natural, and even agricultural, erosion occurring over dozens of years [61]. The results of this study suggested that increasing the GDP-2 proportion of total GDP could worsen soil erosion. A study of the WuLanMuLun River, in the Shenfu mining area, indicated that sediment discharge had doubled after mining commenced, the erosion modulus of the mining area had increased by 15,000 t/km^2^·a, with a 14.965-million ton increase in the annual soil erosion loss [62]. In addition, heavy industry, mining, and construction not only directly influence the land surface through excavation and waste accumulation, but they indirectly further aggravate soil erosion through road transport and the consumption of resources and energy [63].

In general, the urbanization process is complicated. It may promote soil conservation by reducing the soil disturbance driven agricultural activity, as more people migrate to urban areas [32]. However, in contrast to that, secondary industries, especially those in mining, transportation, and construction, which all benefit from rapid urbanization, would have a side effect on soil erosion. Therefore, in the urbanization process in Inner Mongolia, the principles of urban planning should be determined before future construction, with the layout and form of the city optimized to make full use of the urban space. By making full and better use of the opportunities for the human population transfer created by urbanization and industrialization, adjusting the policy for registering urban households, reducing the economic dependence of farmers and herdsmen on ecosystems, and helping people to transfer successfully into urban centers, we could achieve enhanced ecological protection and restoration of land. To minimize the harm to soil that is generated from the development of secondary industries, and to reduce soil erosion while exploiting regional resources, it is necessary to adjust and upgrade the industrial structure, strengthen the management of resources, and improve the efficiency of resource utilization, while adopting a strategy of alternative resource use and environmental protection. Collectively, such action should work to enhance the sustainability of industry, maintain the quality of resources and the environment, and optimize the process of urbanization.

## 6. Conclusions

Inner Mongolia experienced a rapid development stage of urbanization between 2000 and 2010. Its urban population increased by 16.47% from 9.72 million people in 2000 to 11.32 million people in 2010. Over the ten-year period, this urban population grew much faster than rural population (2.52%). In addition, urbanization was closely linked to economic development. The GDP-2 growth rate increased by 29.66% while the GDP-1 rate decreased by 64.87%. At the same time, construction areas and town areas grew substantially, by 33.33% and 31.71%, respectively. Our results, however, also showed there was a significant improvement in soil erosion during the same study period as the area affected by soil erosion decreased from 704,817 km^2^ to 674,135 km^2^, or by 4.35%. From the perspective of soil erosion intensity, the area affected by slight erosion increased by approximately 895 km^2^, whereas the area affected by other grades of soil erosion decreased nearly 31,577 km^2^ with moderate erosion, 7935 km^2^, intense erosion, 2656 km^2^, strong erosion, 10,031 km^2^ and severe erosion, 10,955 km^2^, respectively. Considering the two main types of soil erosion, of the 30,682 km^2^ total area affected, 5817 km^2^ consisted of water erosion and 27,298 km^2^ consisted of wind erosion. Urbanization had particular effects, either positive or negative, on the ten-year change in soil erosion. Urbanization factors together could explain 20.5% of the variation in soil erosion over the period studied. This joint effect arose from the urban population and urbanization rate increasing, and both the rural population size and GDP-2 rate decreasing from 2000 to 2010 in Inner Mongolia.

## Figures and Tables

**Table 1:** Principal data sources.

Data	Resolution	Source
Digital elevation model (DEM)	90 m	Computer Network Information Center (CNIC) of CAS
Soil map and attribute data	1:1,000,000	The second National Soil Survey of China
Ecosystem classification	90 m, TM	Remote Sensing Applications of CAS
Average annual rainfall erosivity	-	Beijing Normal University
Precipitation	0.05 degree	Chinese National Metrological Information Center/China Meteorological Administration (NMIC/CMA)
Temperature	0.05 degree	NMIC/CMA
Wind speed	0.05 degree	The Cold and Arid Regions Science Data Center (CARD)/ The China Meteorological Science Data Service Network
Annual solar radiation data/snow cover	-	The Cold and Arid Regions Sciences Data Center in Lanzhou (http://westdc.westgis.ac.cn)
Vegetation cover	90 m	CAS
Socioeconomic data	Counties	Inner Mongolia Statistical Yearbook

**Table 2:** Changes of urbanization levels between 2000 and 2010 in Inner Mongolia.

Year	2000	2010	2000–2010	
	Total	Total	Difference	Change (%)
Total population (millions)	23.06	25.00	1.94	8.40
Rural population (millions)	13.34	13.68	0.34	2.52
Urban population (millions)	9.72	11.32	1.60	16.47
Urbanization rate (%)	42.16	45.29	3.14	7.44
GDP-1 rate (%)	25.59	8.99	−16.60	–64.87
GDP-2 rate (%)	40.36	52.33	11.97	29.66
Town areas (km^2^)	9800	12,900	3100	31.71
Construction areas (km^2^)	9600	12,800	3200	33.33

GDP-1 (primary industry): agriculture, forestry, animal husbandry and fisheries; GDP-2 (secondary industry): mining, construction industry, and manufacturing [50]; GDP-1 rate: share of total GDP; GDP-2 rate: share of total GDP.

**Table 3:** Land cover conversions in Inner Mongolia between 2000 and 2010.

Transferred Area (km^2^)	Forest	Shrub	Grassland	Wetland	Farmland	Urban Land	Sandy	Bare Land
Forest	-	3.26	13.37	12.94	18.42	39.19	1.00	0.49
Shrub	2.07	-	7.01	0.49	1.62	23.45	0.10	0.68
Grassland	789.00	400.95	-	237.98	679.65	1754.03	165.34	143.65
Wetland	44.42	8.44	565.10	-	568.77	90.49	220.94	122.28
Farmland	147.57	162.53	112.15	237.69	-	432.60	2.89	4.73
Urban land	0.55	6.4	10.00	1.47	5.56	-	0.04	1.10
Sandy	13.97	16.22	173.03	45.63	76.57	41.66	-	2.74
Bare land	21.23	46.02	174.64	162.98	49.25	127.49	0.94	-
Decrease	−88.67	−35.42	−4170.60	−1620.44	−1100.16	−25.12	−369.82	−582.55
Increase	1018.82	643.82	1041.92	686.23	1381.41	2469.73	390.24	275.17
Net change	930.15	608.40	−3128.68	−934.21	281.25	2444.61	20.42	−307.38

**Table 4:** Changes in the areas affected by soil erosion between 2000 and 2010 in Inner Mongolia.

Soil Erosion	Water Erosion				Wind Erosion				Total Erosion Areas			
	2000	2010	2000–2010		2000	2010	2000–2010		2000	2010	2000–2010	
	Area (km^2^)	Area (km^2^)	Variation (km^2^)	Change (%)	Area (km^2^)	Area (km^2^)	Variation (km^2^)	Change (%)	Area (km^2^)	Area (km^2^)	Variation (km^2^)	Change (%)
Slight	106,907	104,105	−2802	−2.62	234,010	238,006	3996	1.71	328,716	329,611	895	0.27
Moderate	15,312	14,007	−1305	−8.52	117,106	110,200	−6906	−5.90	122,864	114,929	−7935	−6.46
Intense	4708	3806	−902	−19.16	60,417	57,523	−2894	−4.79	63,411	60,755	−2656	−4.19
Strong	2405	1800	−605	−25.16	121,201	111,306	−9895	−8.16	110,900	100,869	−10,031	−9.05
Severe	1309	1106	−203	−15.51	94,110	82,511	−11,599	−12.32	78,926	67,971	−10,955	−13.88
Sum	130,641	124,824	−5817	−4.45	626,844	599,546	−27,298	−4.35	704,817	674,135	−30,682	−4.35

**Table 5:** The correlation coefficients between urbanization and soil erosion.

Change of Intensity (*n* = 101)	Change of Impact Factor			
	GDP-2 Rate	Urban Population	Rural Population	Urbanization Rate
Water erosion	0.393 *	−0.308 *	0.353 **	−0.318 *
Wind erosion	0.993 **	−0.420 **	0.285 *	−0.442 **

* *p* < 0.05, ** *p* < 0.01.

## References

[1] Martine G., Marshall A. (2007). State of world population 2007: Unleashing the potential of urban growth. State of World Population: Unleashing the Potential of Urban Growth.

[2] Yan X., Lin Z. (2004). The change of spatial disparities of urban development in China, 1990s. Acta Geogr. Sin..

[3] Kirkby R.J. (2018). Urbanization in China: Town and Country in a Developing Economy 1949–2000 ad.

[4] Shin H.B. (2015). Urbanization in China. International Encyclopedia of the Social and Behavioral Sciences.

[5] Zhao J., Liu H., Dong R. (2008). Sustainable urban development: Policy framework for sustainable consumption and production. Int. J. Sustain. Dev. World Ecol..

[6] Adamec R.E. (1976). The interaction of hunger and preying in the domestic cat (felis catus): An adaptive hierarchy?. Behav. Biol..

[7] Ghani S.E., Kanbur R. (2013). Urbanization and (in) Formalization.

[8] Hugo G. (2017). New Forms of Urbanization: Beyond the Urban-Rural Dichotomy.

[9] Hammond A.L. (1992). World Resources: 1992-93; A Guide to the Global Environment.

[10] Sklyarova E.K. (2016). Comparative aspects of scientific research of the urbanization problem. Научный альманах стран Причерноморья.

[11] Jin D., Gu S., Shen L. (2004). Analysis on urbanization dynamics: On factors and strategic choices of urbanization in China. China Popul. Resour. Environ..

[12] Faulkner S. (2004). Urbanization impacts on the structure and function of forested wetlands. Urban Ecosyst..

[13] McDonald R.I. (2008). Global urbanization: Can ecologists identify a sustainable way forward?. Front. Ecol. Environ..

[14] Grimm N.B., Foster D., Groffman P., Grove J.M., Hopkinson C.S., Nadelhoffer K.J., Pataki D.E., Peters D.P. (2008). The changing landscape: Ecosystem responses to urbanization and pollution across climatic and societal gradients. Front. Ecol. Environ..

[15] Deng X., Huang J., Rozelle S., Uchida E. (2006). Cultivated land conversion and potential agricultural productivity in China. Land Use Policy.

[16] Gardi C., Panagos P., Van Liedekerke M., Bosco C., De Brogniez D. (2015). Land take and food security: Assessment of land take on the agricultural production in Europe. J. Environ. Plan. Manag..

[17] Pandey B., Seto K.C. (2015). Urbanization and agricultural land loss in India: Comparing satellite estimates with census data. J. Environ. Manag..

[18] Bao C., Fang C. (2012). Water resources flows related to urbanization in China: Challenges and perspectives for water management and urban development. Water Resour. Manag..

[19] Pimentel D., Harvey C., Resosudarmo P., Sinclair K., Kurz D., McNair M., Crist S., Shpritz L., Fitton L., Saffouri R. (1995). Environmental and economic costs of soil erosion and conservation benefits. Science.

[20] Rao E., Xiao Y., Ouyang Z., Yu X. (2015). National assessment of soil erosion and its spatial patterns in China. Ecosyst. Health Sustain..

[21] Chen L. (2012). Implement the Central Decision and Deployment Thoroughly, and Write a New Chapter of Soil and Water Conservation and Ecological Construction with Chinese Characteristics.

[22] Otani S., Kurosaki Y., Kurozawa Y., Shinoda M. (2017). Dust storms from degraded drylands of Asia: Dynamics and health impacts. Land.

[23] Goudie A.S. (2014). Desert dust and human health disorders. Environ. Int..

[24] Copeland N., Sharratt B., Wu J., Foltz R., Dooley J. (2009). A wood-strand material for wind erosion control: Effects on total sediment loss, PM 10 vertical flux, and PM 10 loss. J. Environ. Qual..

[25] Yang G., Ming Q. (2007). Pressures of urbanization on ecology and environment in Inner Mongolia. Arid Land Geogr..

[26] Qiao B., Fang C. (2005). The dynamic coupling model of the harmonious development between urbanization and eco-environment and its application in arid area. Acta Ecol. Sin..

[27] Ye D., Chou J., Liu J. (2000). Causes of sand-stormy weather in northern China and control measures. Acta Geogr. Sin..

[28] Tao W., Chen G., Qian Z., Yang G., Qu J., Li D. (2001). Situation of sand-dust storms and countermeasures in north China. J. Desert Res..

[29] Li L., Guo Q. (2001). The parsing of sandstorm source in Beijing in 2000. Res. Environ. Sci..

[30] Jiang L., Xiao Y., Zheng H., Ouyang Z. (2016). Spatio-temporal variation of wind erosion in Inner Mongolia of China between 2001 and 2010. Chin. Geogr. Sci..

[31] Ouyang Z., Zheng H., Xiao Y., Polasky S., Liu J., Xu W., Wang Q., Zhang L., Xiao Y., Rao E. (2016). Improvements in ecosystem services from investments in natural capital. Science.

[32] Rao E., Xiao Y., Ouyang Z., Zheng H. (2016). Changes in ecosystem service of soil conservation between 2000 and 2010 and its driving factors in southwestern China. Chin. Geogr. Sci..

[33] Renard K.G. (1997). Predicting Soil Erosion by Water: A Guide to Conservation Planning with the Revised Universal Soil Loss Equation (RUSLE).

[34] Chen T., Niu R., Li P., Zhang L., Du B. (2011). Regional soil erosion risk mapping using RUSLE, GIS, and remote sensing: A case study in Miyun Watershed, north China. Environ. Earth Sci..

[35] Demirci A., Karaburun A. (2012). Estimation of soil erosion using RUSLE in a GIS framework: A case study in the Buyukcekmece Lake Watershed, northwest Turkey. Environ. Earth Sci..

[36] Zobeck T.M., Pelt S.V., Stout J.E., Popham T.W., Ascough Ii J.C., Flanagan D.C. (2001). Validation of the Revised Wind Erosion Equation (REWQ) for single events and discrete periods. Soil Erosion Research for the 21th Century.

[37] Borrelli P., Lugato E., Montanarella L., Panagos P. (2017). A new assessment of soil loss due to wind erosion in European agricultural soils using a quantitative spatially distributed modelling approach. Land Degrad. Dev..

[38] Panagos P., Borrelli P., Poesen J., Ballabio C., Lugato E., Meusburger K., Montanarella L., Alewell C. (2015). The new assessment of soil loss by water erosion in Europe. Environ. Sci. Policy.

[39] Wischmeier W.H., Smith D.D. (1978). Predicting Rainfall Erosion Losses—A Guide to Conservation Planning.

[40] State Council Office of China (SCO) (2010). Training Manual of the First Nationwide Water Resources Survey: Census of Soil and Water Conservation.

[41] Zhang K., Shu A., Xu X., Yang Q., Yu B. (2008). Soil erodibility and its estimation for agricultural soils in China. J. Arid Environ..

[42] Van Remortel R.D., Hamilton M.E., Hickey R.J. (2001). Estimating the LS factor for RUSLE through iterative slope length processing of digital elevation data within Arclnfo grid. Cartography.

[43] Chepil W. (1962). A compact rotary sieve and the importance of dry sieving in physical soil analysis 1. Soil Sci. Soc. Am. J..

[44] Zobeck T. (1991). Abrasion of crusted soils: Influence of abrader flux and soil properties. Soil Sci. Soc. Am. J..

[45] Bilbro J., Fryrear D. (1994). Wind erosion losses as related to plant silhouette and soil cover. Agron. J..

[46] Ministry of Water Resources of the People’s Republic of China (MWRPRC) (2008). Standards for Classification and Gradation of Soil Erosion (SL190–2007).

[47] Henderson V. (2003). The urbanization process and economic growth: The so-what question. J. Econ. Growth.

[48] Heilig G.K. (2012). World Urbanization Prospects: The 2011 Revision.

[49] Li X., Fang C., Huang J., Mao H. (2003). The urban land use transformations and associated effects on eco-environment in northwest China arid region: A case study in Hexi region, Gansu province. Quat. Sci..

[50] Standardization Administration of the People’s Republic of China The Classification for National Economic Activities (GB/T 4754-2017). http://www.sac.gov.cn/was5/web/search?channelid=97779&templet=gjcxjg_detail.jsp&searchword=standard_code=%27gb/t%204754-2017%27.

[51] Hou Y., Zhang Y., Wu B. (2013). Eco-environmentalimpact of urbanization process in a sandy area. Bull. Soil Water Conserv..

[52] Chen D. (2004). Introduction the ecological roles of urbanization in desertification area at north of Shaanxi province. Ecol. Econ..

[53] Liu A., Ci L. (1997). A systematic analysis of theman-made influence in modern desertification process. J. Nat. Resour..

[54] Schiettecatte W., Cornelis W., Acosta M., Leal Z., Lauwers N., Almoza Y., Alonso G., Díaz J., Ruíz M., Gabriels D. (2008). Influence of landuse on soil erosion risk in the Cuyaguateje watershed (Cuba). Catena.

[55] Montgomery D.R. (2007). Soil erosion and agricultural sustainability. Proc. Natl. Acad. Sci. USA.

[56] Yang D., Kanae S., Oki T., Koike T., Musiake K. (2003). Global potential soil erosion with reference to land use and climate changes. Hydrol. Process..

[57] Borrelli P., Robinson D.A., Fleischer L.R., Lugato E., Ballabio C., Alewell C., Meusburger K., Modugno S., Schütt B., Ferro V. (2017). An assessment of the global impact of 21st century land use change on soil erosion. Nat. Commun..

[58] Liu S., Wang T. (2012). Climate change and local adaptation strategies in the middle Inner Mongolia, northern China. Environ. Earth Sci..

[59] Chu C., Meng H. (2006). Ecology migration and sustainable development of economy in Inner Mongolia Autonomous region. Res. Agric. Mod..

[60] Wang H., Xu J., Yan M. (2011). Effect of socio-economic factors on soil erosion: A literature review. Prog. Geogr..

[61] Tang K. (2004). Soil and Water Conservation in China.

[62] Zhang H., Wang Z. (1994). The influence of Shenfu-Dongsheng coal mining on river bed siltation and sediment load of Wulanmulun River. Res. Soil Water Conserv..

[63] Feng J., Rui L. (1994). Effects of mining construction on social-economics in Shenfu-Dongsheng coal mine area-an analysis of social-economics changes in Daliuta area. Res. Soil Water Conserv..

